# Harnessing genomics approaches for heat stress resilience in wheat: from discovery to deployment

**DOI:** 10.1093/jxb/erag226

**Published:** 2026-05-18

**Authors:** Annapurna Chitikineni, Farkhandah Jan, Dinesh Kumar Saini, Mukesh Rathore, Dion Bennett, Zhong-Hua Chen, Richard Harper, Liu Xin, Rajeev K Varshney, Reyazul Rouf Mir

**Affiliations:** WA State Agricultural Biotechnology Centre, Centre for Crop and Food Innovation, Food Futures Institute, Murdoch University, Murdoch, WA 6150, Australia; Division of Genetics & Plant Breeding, Faculty of Agriculture, SKUAST-Kashmir, Wadura Campus, J&K, Sopore-193201, Kashmir, India; Department of Plant and Soil Science, Texas Tech University, Lubbock, TX 79409, USA; Division of Genetics & Plant Breeding, Faculty of Agriculture, SKUAST-Kashmir, Wadura Campus, J&K, Sopore-193201, Kashmir, India; Australian Grain Technologies (AGT), Northam, WA 6401, Australia; School of Agriculture, Food and Wine, Faculty of Sciences, Engineering and Technology, The University of Adelaide, Australia; School of Agricultural Science, Centre for Crop and Food Innovation, Food Futures Institute, Murdoch University, Murdoch, WA 6150, Australia; BGI-Shenzhen, Shenzhen, China; WA State Agricultural Biotechnology Centre, Centre for Crop and Food Innovation, Food Futures Institute, Murdoch University, Murdoch, WA 6150, Australia; WA State Agricultural Biotechnology Centre, Centre for Crop and Food Innovation, Food Futures Institute, Murdoch University, Murdoch, WA 6150, Australia; Division of Genetics & Plant Breeding, Faculty of Agriculture, SKUAST-Kashmir, Wadura Campus, J&K, Sopore-193201, Kashmir, India; The New Zealand Institute for Plant and Food Research Limited, New Zealand

**Keywords:** Epigenetic regulation, gene editing, genetic resources, heat stress tolerance, multi-omics, QTL mapping, wheat

## Abstract

Global warming poses a critical threat to wheat (*Triticum aestivum* L.) production, particularly during sensitive reproductive stages. This review synthesizes current understanding of how heat stress affects wheat and the adaptive mechanisms that confer tolerance, with emphasis on recent advances in genomics and biotechnology. Heat stress impairs morphological, physiological, biochemical, and molecular processes, reducing photosynthetic efficiency, accelerating senescence, disrupting assimilate partitioning, and damaging cellular structures. Adaptive responses include optimized water relations, antioxidant defences, osmolyte accumulation, heat shock protein induction, and hormonal regulation, all coordinated by complex gene networks. Advances in genetic dissection through quantitative trait locus (QTL) mapping, genome-wide association studies, and candidate gene discovery have identified loci and alleles linked to thermotolerance. Multi-omics integration has uncovered regulatory pathways, transcription factors, and epigenetic mechanisms, including stress memory, that underpin resilience. Emerging tools such as genome sequencing, pangenomics, genomic prediction, haplotype-informed breeding, and CRISPR-based editing are accelerating the translation of these discoveries into improved cultivars. In addition to summarizing these advances, this review highlights key challenges, from harmonizing heat stress phenotyping and validating causal variants to integrating multi-omics into breeding pipelines, and proposes targeted strategies to bridge the gap between discovery and deployment to enable the development of climate-ready wheat cultivars.

## Teaser

This review integrates recent advances in genomics, multi-omics, and biotechnology with a critical assessment of key challenges, providing a roadmap for accelerating development of heat-resilient wheat cultivars in the face of global warming.

## Introduction

Wheat (*Triticum aestivum* L.) is the nutritional cornerstone for >35% of the global population, supplying calories and protein to >2.5 billion people (Sharma and Sharma, 2025). Its adaptability to diverse agro-climatic zones, from temperate to semi-arid and subtropical regions, has enabled widespread cultivation worldwide (Zhai *et al.*, 2021; Kheiri *et al.*, 2024). However, climate change, particularly rising global temperatures, poses serious threats to wheat production (Shewry, 2024). Among abiotic stresses, heat stress is a major yield-limiting factor, especially during reproductive stages such as anthesis and grain filling (Chen *et al.*, 2022).

Plant responses to high-temperature stress have been widely studied in both model and crop species, revealing conserved physiological, biochemical, and molecular mechanisms underlying thermotolerance (Zhao *et al.*, 2017). Studies in *Arabidopsis thaliana*, rice (*Oryza sativa*), maize (*Zea mays*), and other plants show that heat stress disrupts photosynthesis, cellular homeostasis, and redox balance while activating protective pathways involving heat-shock proteins (HSPs), heat-shock transcription factors (HSFs), hormonal signalling, and antioxidant systems (Balfagón *et al.*, 2020; Janni *et al.*, 2020; Kan *et al.*, 2023; Kumar *et al.*, 2025). Central regulatory pathways such as Ca^2+^ signalling and mitogen-activated protein kinase (MAPK) cascades, and hormone-mediated responses involving abscisic acid (ABA) and ethylene are broadly conserved and coordinate cellular protection against thermal damage (Kumar *et al.*, 2025). In natural environments, high temperature often coincides with other stresses such as drought or high irradiance, requiring coordinated stress–response networks that integrate multiple signalling pathways (Balfagón *et al.*, 2020; Zhao *et al.*, 2020).

Comparative studies indicate that many crops share similar reproductive vulnerabilities under heat stress. Elevated temperatures during flowering impair pollen viability, anther dehiscence, and fertilization in Arabidopsis, rice, maize, and wheat, leading to substantial yield losses (Zhao *et al.*, 2017). Nevertheless, important physiological differences exist among species. Cool-season cereals such as wheat are particularly sensitive to elevated temperatures, with germination and early growth declining above ∼25 °C, whereas warm-season C_4_ crops such as maize maintain higher photosynthetic rates under elevated temperatures than C_3_ crops (Sage and Kubien, 2007). At the molecular level, the heat-shock response is largely conserved across species through HSF-mediated activation of HSPs. However, species-specific regulatory mechanisms have also been identified. For instance, detailed HSF regulatory networks and thermo-memory mechanisms have been characterized in Arabidopsis (Dündar *et al.*, 2025), while rice studies have identified numerous quantitative trait loci (QTLs) associated with spikelet fertility under heat stress (Nubankoh *et al.*, 2020), and maize research highlights the sensitivity of chloroplast structure and photosynthetic machinery to elevated temperatures, with heat stress disrupting chloroplast integrity and photosystem function (Li *et al.*, 2020; El-Sappah *et al.*, 2022). In comparison, research progress in wheat has historically been slower due to its large and complex hexaploid genome (∼16–17 Gb), limited mutant resources, and strong genotype x environment (G x E) interactions (Yao *et al.*, 2025). Nevertheless, recent advances in genome sequencing, multi-omics, and high-throughput phenotyping are accelerating the discovery of heat-responsive genes and pathways in wheat.

Global mean temperatures have already increased by ∼1.1 °C since 1900, and each additional 1 °C rise is projected to reduce wheat yields by 4–6% (Zampieri *et al.*, 2017). The threat is particularly severe in South Asia, sub-Saharan Africa, and parts of the Mediterranean (Jaradat, 2017; Chen *et al.*, 2022). Increasing heatwaves (>35 °C for ≥3 d), often combined with elevated CO_2_ and drought, further exacerbate yield losses (Ahmad *et al.*, 2025). Heat stress reduces photosynthesis by limiting Rubisco activation, accelerates crop development and maturity, and lowers grain quality through adverse effects on grain protein composition, including glutenin accumulation (Tricker *et al.*, 2018). High-temperature stress is broadly categorized as mild or moderate heat stress, involving prolonged exposure to moderately elevated temperatures, and severe heat stress, resulting from short episodes of extreme temperatures (Wahid *et al.*, 2007). In wheat-growing environments, terminal heat stress during reproductive stages, particularly anthesis and grain filling, is the most common and damaging form, as brief exposure to temperatures >30–35 °C can reduce grain set, shorten grain-filling duration, and ultimately decrease yield. These stage-specific sensitivities, together with adaptation of wheat to cooler climates, make heat stress a major constraint on stable wheat production under current and future climates (Farooq *et al.*, 2011).

To counter these effects, wheat deploys a range of morphological, physiological, biochemical, and molecular responses. These include leaf rolling, altered leaf orientation, and accelerated maturity (Choudhary *et al.*, 2022); regulation of transpiration, membrane stability, and stomatal conductance (Bala *et al.*, 2014); accumulation of antioxidants such as α-tocopherol and flavonoids (Zahra *et al.*, 2023); and induction of HSPs and stress-responsive genes that stabilize proteins and membranes (Mittler *et al.*, 2011). Because heat tolerance is highly polygenic, advanced genomics and breeding tools are essential for improving thermotolerance. Recent advances include identification of heat-responsive genes, QTLs, transcription factors (TFs), and molecular markers (Guan *et al.*, 2018; Sall *et al.*, 2018). Functional genomics approaches such as CRISPR/Cas9 [clustered regularly interspaced palindromic repeats (CRISPR)/CRISPR-associated protein 9] are increasingly being applied; for example, editing dehydration-responsive element-binding protein 3 (*TaDREB3*) has been identified as a promising target for enhancing heat/drought tolerance in wheat (Rai *et al.*, 2023). Despite these advances, important knowledge gaps remain. Multi-environment testing is needed to better link genotype–phenotype relationships, while the molecular networks underlying thermotolerance under combined stresses remain poorly understood. Multi-omics datasets are still underutilized, and functional validation of candidate genes remains limited. Moreover, most studies evaluate heat stress in isolation, overlooking the complex stress combinations encountered under field conditions (Harfouche *et al.*, 2019).

**Fig. 1.**
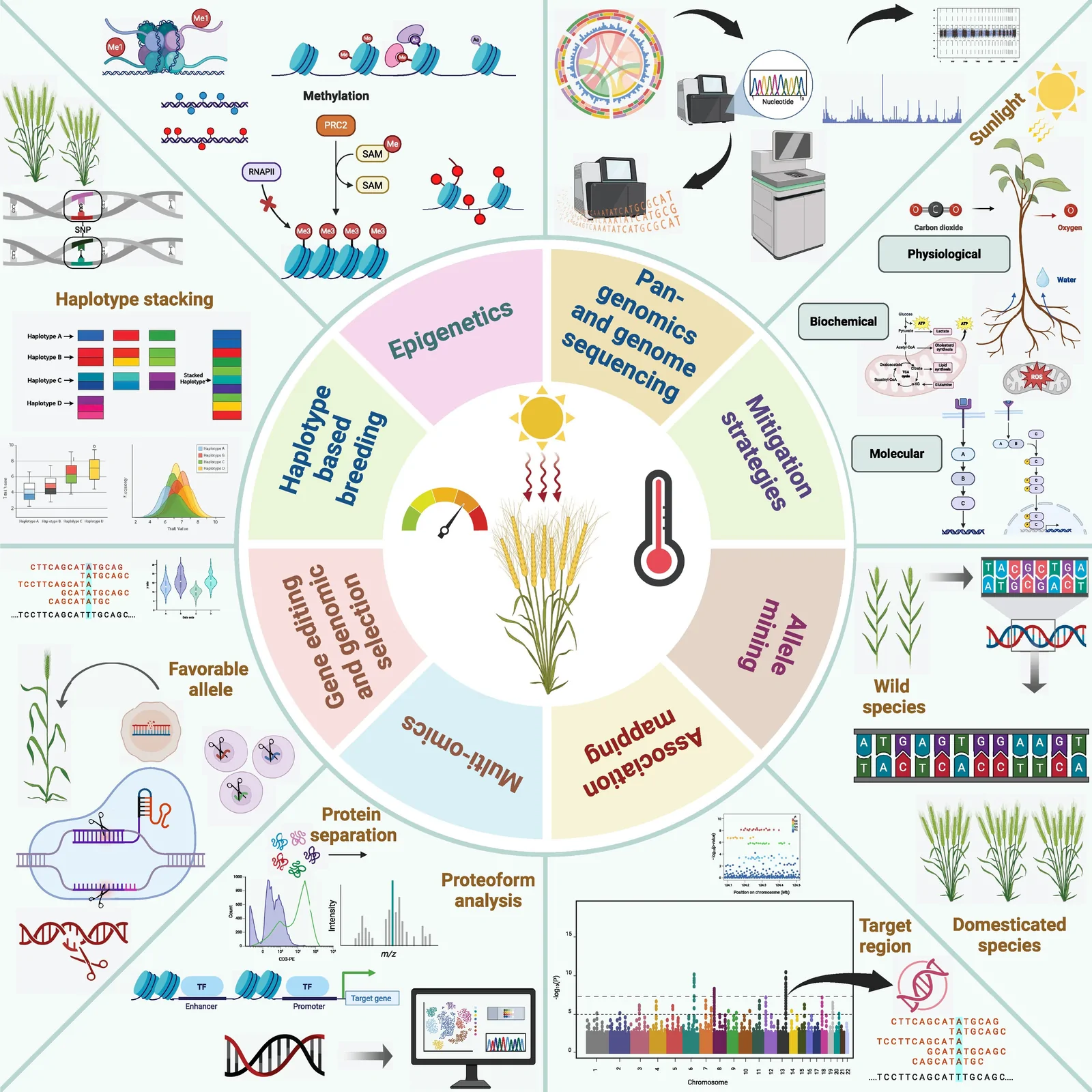
Integrated framework of genomic approaches and mechanisms for enhancing heat tolerance in wheat. This figure presents a comprehensive overview of the various genomic tools, molecular mechanisms, and breeding strategies employed to improve heat stress tolerance in wheat. The figure illustrates recent advances and strategies employed to understand and improve heat stress tolerance. Approaches include epigenetics (DNA methylation and chromatin remodeling), pan-genomics and genome sequencing for deeper genetic insights, and mitigation strategies such as physiological, biochemical, and molecular responses. Allele mining explores beneficial alleles from wild relatives, while association mapping identifies genomic regions linked to stress tolerance traits. Multi-omics approaches (transcriptomics, proteomics, and metabolomics) unravel complex regulatory networks. Gene editing enables precise modification of stress-related genes, and genomic prediction and haplotype-based breeding accelerate the development of heat-resilient varieties. Together, these interdisciplinary strategies contribute to deciphering and harnessing the genetic architecture of heat tolerance in crops. Created in BioRender. Chitikineni, A. (2026) https://BioRender.com/00mgupm.

This review highlights the urgent need to safeguard wheat productivity under intensifying heat episodes. We synthesize adaptive mechanisms across physiological, biochemical, and molecular levels and connect them with discovery approaches such as QTL mapping, genome-wide association study (GWAS), and pan-genomics. We also discuss enabling technologies, including genomic prediction, haplotype-assisted breeding, and CRISPR-based genome editing, within an integrative framework (Fig. 1). Key research gaps, including reproductive-stage thermotolerance, combined-stress genetics, and limited field validation, are identified, and integrative strategies combining harmonized phenotyping, multi-omics integration, and targeted genome editing are proposed to accelerate the development of climate-resilient, heat-tolerant wheat cultivars.

## Heat stress impacts and adaptive mechanisms in wheat

### Heat-induced morphological and phenological changes

**Fig. 2.**
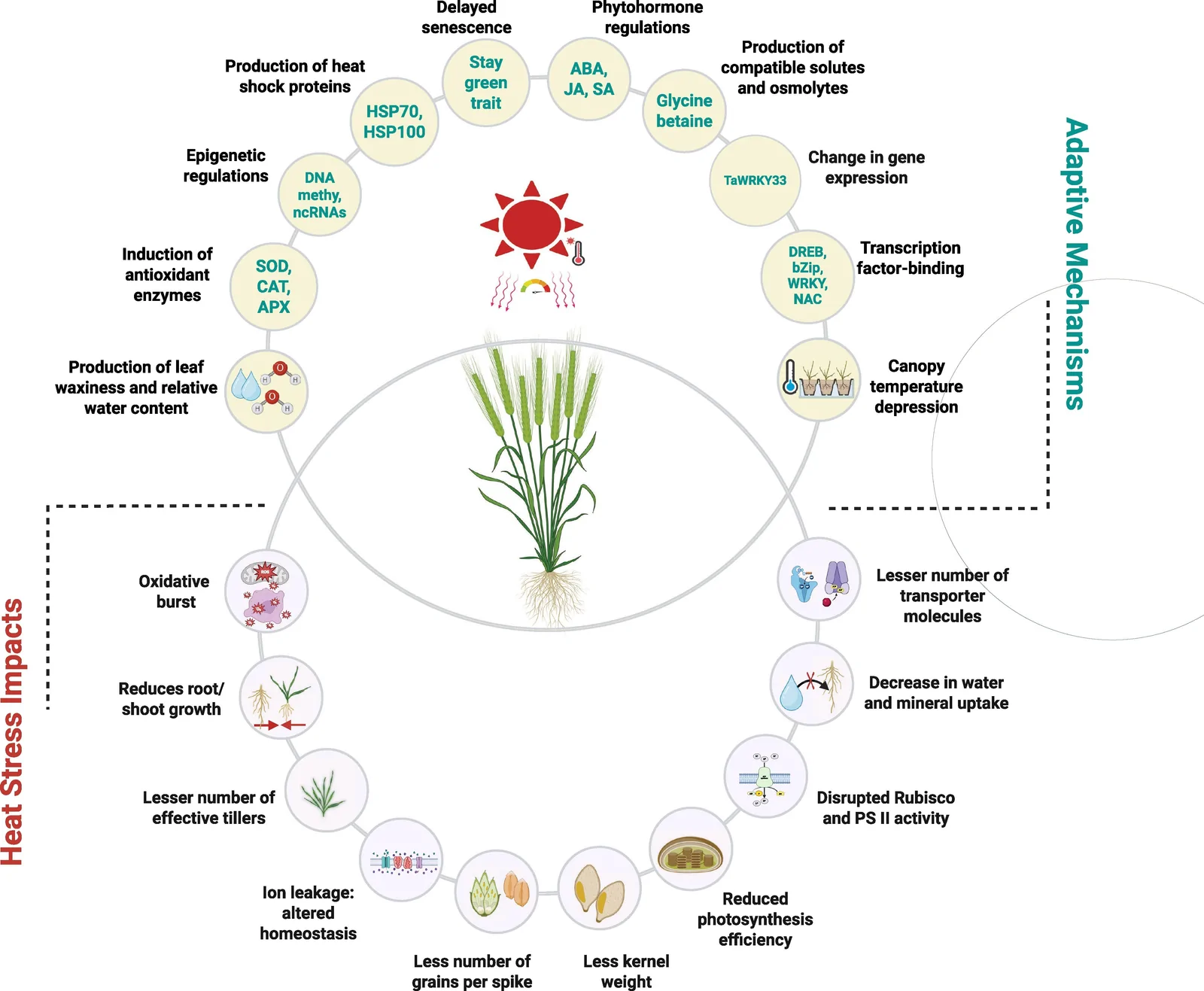
Effects of heat stress and tolerance mechanisms of wheat. This figure illustrates the contrasting responses of wheat to heat stress, highlighting both the detrimental impacts (bottom side) and the adaptive mechanisms (upper side) employed by the plant to mitigate heat stress. The detrimental impacts of heat stress include oxidative burst, reduced root and shoot growth, ion leakage, fewer grains per spike, lower kernel weight, and impaired photosynthetic efficiency. Plants mitigate heat stress through key tolerance mechanisms including production of heat shock proteins (e.g. HSP70 and HSP100), activation of antioxidant enzymes (SOD, CAT, and APX), epigenetic regulation, delayed senescence traits (stay-green), phytohormone adjustments (ABA, JA, and SA), accumulation of osmolytes (e.g. glycine betaine), transcription factor activation (DREB, WRKY, and NAC) and maintenance of canopy temperature depression. Collectively, these physiological, biochemical, and molecular responses enhance heat resilience and protect crop productivity under high-temperature stress. Created in BioRender. Chitikineni, A. (2026) https://BioRender.com/vedky56.

High temperature adversely affects wheat from germination to grain filling, with severity determined by the timing, intensity, and duration of stress, as well as the developmental stage at exposure (Kumar *et al.*, 2022). During germination and seedling growth, extreme temperature (e.g. 45 °C for 2 h at 7 d after germination) sharply reduces shoot and root dry mass and length, impairing establishment and limiting yield potential (Gupta *et al.*, 2013). At the vegetative stage, heat stress shortens growth duration, reduces leaf area and root and shoot biomass, and decreases effective tiller number, thereby restricting spike initiation and yield capacity (Yadav *et al.*, 2022). The reproductive stage is most vulnerable to heat stress, which can substantially reduce biomass accumulation and yield depending on genotype and stress severity (Sharma *et al.*, 2015). Stress at this stage accelerates leaf senescence, lowers grain number and kernel weight, and irreversibly damages reproductive organs, causing major yield and quality losses (Fig. 2) (Pradhan *et al.*, 2012a; Weldearegay *et al.*, 2012). Heat stress further accelerates heading, anthesis, and physiological maturity, shortening grain filling by up to 39% and reducing yields >50% under severe stress (Groli *et al.*, 2024). Such morphological and phenological alterations are central to heat-induced yield loss. However, traits such as tiller survival, flag leaf longevity, and grain-filling dynamics remain underexplored in multi-environment field settings. Greater integration of these traits into breeding indices and crop models could enhance selection for heat resilience. Gaps also persist in identifying early-season traits that predict terminal heat tolerance. Root traits, for instance, remain less studied than above-ground features, yet evidence shows that heat-tolerant seedlings maintaining root length and biomass under early stress often yield better as adults. This highlights early root vigour as a promising but underutilized selection target.

### Physiological adaptations

At the physiological level, heat stress in wheat reduces leaf water potential and relative water content (RWC), particularly in sensitive genotypes, impairing water uptake and photosynthetic efficiency (Fig. 2) (Almeselmani *et al.*, 2009). Tolerant genotypes mitigate these effects through traits such as higher RWC, better stomatal conductance, leaf waxiness, altered leaf orientation, and deeper roots, which enhance transpirational cooling and lower canopy temperature, positively linked to yield (Sarkar *et al.*, 2021). Photosynthesis is especially vulnerable, with damage to PSII, altered chloroplast ultrastructure, reduced Rubisco activity, and consequent chlorophyll degradation leading to decreased carbon assimilation (Monneveux *et al.*, 2003). In susceptible genotypes, respiration surpasses photosynthesis, causing biomass loss and energy waste via increased photorespiration (Abasi *et al.*, 2024). Heat stress also disrupts nutrient uptake, particularly nitrogen, phosphorus, and potassium, weakening antioxidant defences and triggering oxidative stress (Klimenko *et al.*, 2006).

Despite the recognized importance of traits such as canopy temperature depression (CTD) and stay-green, their expression is strongly influenced by developmental stage and environment, complicating reliable selection. Although screening approaches such as late sowing, heat tents, and CTD/SPAD/*F*_v_/*F*_m_ measurements are widely used, there is no globally standardized physiological protocol, and debate continues over which trait sets or indices best capture resilience. This heterogeneity hampers comparative breeding progress. Moreover, while early reproductive heat responses are well studied, the physiological processes underlying heat-accelerated senescence and grain size reduction during grain filling and maturation remain poorly resolved, representing a key research gap.

### Biochemical adaptations

A major biochemical consequence of heat stress is excessive production of reactive oxygen species (ROS), including superoxide radicals, hydrogen peroxide, and hydroxyl radicals, arising from over-reduction of mitochondrial and chloroplast electron transport chains (Fig. 2) (Mittler *et al.*, 2022). At low levels, ROS act as signalling molecules, but in excess they damage lipids, proteins, and nucleic acids, impairing cellular function and accelerating senescence (Jan *et al.*, 2025). To counter this, wheat activates enzymatic antioxidants (Gill *et al.*, 2015; Khanzada *et al.*, 2025), which detoxify ROS and maintain redox balance. Proline accumulation is another defence, especially during the vegetative stage at temperatures >25 °C. However, under heat shock (30–40 °C for 2 h), proline declines as ROS production overwhelms scavenging capacity, causing membrane damage (Kumar *et al.*, 2013). Other osmolytes such as glycine betaine and sugars stabilize membranes, preserve proteins, and maintain osmotic balance.

HSPs (e.g. HSP20, HSP60, HSP70, HSP90, and HSP100) act as molecular chaperones, rapidly induced to refold denatured proteins, prevent aggregation, and stabilize complexes, thereby preserving proteostasis and sustaining metabolism under heat stress (Wang *et al.*, 2004). Among these, HSP70 and HSP90 are particularly critical in maintaining structural integrity and supporting recovery after thermal damage (Dubrez *et al.*, 2020). Similarly, overexpression of the *BADH* gene enhances glycine betaine accumulation, which protects photosynthesis, maintains leaf water status, and strengthens antioxidant defences, contributing to yield stability under heat and drought (Wang *et al.*, 2010). Collectively, antioxidants, osmolytes, and HSPs buffer against oxidative stress, membrane damage, and metabolic imbalance, but their coordination across tissues and stages remains poorly understood, limiting the identification of robust biochemical markers for field-level resilience.

Hormonal regulation also plays a pivotal role in protection. ABA reduces transpirational water loss via stomatal closure and activates stress-responsive genes (Bharath *et al.*, 2021). Grain ABA concentration and exogenous ABA enhances grain filling and sink capacity (Yang *et al.*, 2014). Salicylic acid (SA), ethylene, and jasmonic acid (JA) activate antioxidant and defence pathways, mitigating oxidative and cellular damage (Prerostova *et al.*, 2020). Auxins and cytokinins delay heat-induced senescence, sustaining assimilate supply and nutrient remobilization (Prerostová and Vanková, 2023), while gibberellins may promote growth depending on the developmental stage (Guo *et al.*, 2022). Hormonal crosstalk integrates biochemical defences with physiological adjustments such as canopy cooling, sustained photosynthesis, and improved reproductive resilience (Sehar *et al.*, 2023). However, the molecular links connecting hormonal, antioxidant, and chaperone systems remain unclear, particularly during grain filling when yield losses are greatest.

### Molecular adaptations

Heat stress perception in wheat begins at the plasma membrane, where changes in lipid fluidity and protein conformation activate heat-sensitive Ca^2+^ channels (Abdelrahman *et al.*, 2020). The resulting Ca^2+^ influx engages Ca^2+^-binding proteins and secondary messengers such as ROS and nitric oxide, which activate MAPKs and calcium-dependent protein kinases (CDPKs) (Raja *et al.*, 2017). These cascades relay stress cues to the nucleus, driving transcriptional reprogramming of heat-responsive genes including HSPs, antioxidants, and osmoprotectants (Fig. 2) (Xue *et al.*, 2015). Epigenetic processes, such as DNA methylation, histone modifications, and chromatin remodelling, further regulate stress gene accessibility and contribute to heat stress memory (Gardiner *et al.*, 2015). Non-coding RNAs [miRNAs, siRNAs, and long non-coding RNAs (lncRNAs)] also modulate mRNA stability and translation; for instance, specific wheat miRNAs target transcripts encoding TFs, signalling proteins, and enzymes essential for adaptation (Ragupathy *et al.*, 2016; Ravichandran *et al.*, 2019) (see ‘Advanced molecular strategies’).

Together, these molecular systems integrate environmental signals with physiological and biochemical responses to enable rapid heat stress adaptation. However, key gaps remain. The master regulators coordinating tolerance pathways are still unclear, and many candidate TFs lack functional validation in wheat. Most studies focus on seedlings or short-term heat treatments, leaving reproductive stages such as flowering and grain filling poorly characterized under field conditions. Identifying molecular triggers of floret sterility and gene networks that sustain grain weight under prolonged heat stress will be critical for translating molecular insights into durable field-level thermotolerance.

## Genetic resources and germplasm utilization

The adverse effects of heat stress on wheat production are amplified by the increasing genetic uniformity of modern cultivars, particularly in developed countries (Fu *et al.*, 2015). This narrowing genetic base highlights the need to harness novel genetic resources and adaptive traits to counter yield losses. Globally, ∼ 0.8 million wheat genetic resources, including landraces, wild relatives, and gene bank accessions, are conserved in germplasm collections, offering a vast reservoir of alleles for stress tolerance (Dempewolf *et al.*, 2014; Sharma *et al.*, 2021). A well-known example is the A. E. Watkins landrace collection, assembled in the 1930s from landrace cultivars originating from >30 countries, which represents a highly diverse resource for gene discovery and allele mining in bread wheat (Wingen *et al.*, 2014). Several case studies have demonstrated the successful utilization of these resources in breeding programmes (Adhikari *et al.*, 2022; Rasheed *et al.*, 2025). Landraces and resynthesized hexaploid wheat are particularly valuable due to their broad genomic variation and adaptation to harsh environments (Trethowan and Mujeeb-Kazi, 2008). Many modern stress-tolerant cultivars trace part of their ancestry to landrace backgrounds; for example, the Spanish variety ‘Aragon 03’ originated from a native landrace with strong resilience (Royo and Briceño-Félix, 2011).

Historical breeding bottlenecks and strong selection pressure have reduced genetic variation in modern wheat, particularly within the D genome (Farooq *et al.*, 2026). To address this limitation, CIMMYT and other breeding programmes have incorporated exotic germplasm into pre-breeding pipelines, with landraces and primary synthetic wheats serving as key donors of novel diversity (Reynolds *et al.*, 2007; Sehgal *et al.*, 2015; Singh *et al.*, 2018). Primary synthetics, produced by crossing durum wheat with the D-genome donor species *Aegilops tauschii*, have contributed valuable alleles for disease resistance as well as drought and heat tolerance (Trethowan and Mujeeb-Kazi, 2008; Aberkane *et al.*, 2021). Multiple synthetic derivatives (MSDs) developed from these materials have shown resilience under continuous heat stress, with backcross inbred lines (BILs) derived from them producing several promising heat-tolerant lines (Elbashir *et al.*, 2017; Gorafi *et al.*, 2018; Itam *et al.*, 2021). The value of exotic introgressions is further illustrated by the High Biomass Association Mapping Panel, which includes 149 spring wheat lines containing 0.5–43% exotic donor contributions, including chromosomal segments from *Thinopyrum ponticum* (7D) and *Secale cereale* (1B) (Molero *et al.*, 2019; Joynson *et al.*, 2021). Three exotic-derived loci identified in this panel were associated with yield gains exceeding 50% and canopy cooling of ∼2 °C under heat stress. Complementary physiological pre-breeding efforts have also screened nearly 60 000 accessions from the World Wheat Collection to develop trait-specific panels for marker discovery and breeding applications (Sukumaran *et al.*, 2021).

Wild relatives remain another critical source of adaptive variation. Wild emmer wheat (*Triticum turgidum* ssp. *dicoccoides*) has contributed heat tolerance alleles through introgression into elite germplasm (Peng *et al.*, 2012; Ullah *et al.*, 2021). Other wild relatives, including *Triticum urartu*, *Aegilops speltoides*, and *Aegilops geniculata*, provide valuable traits such as membrane thermostability, deep rooting, enhanced antioxidant capacity, and stay-green characteristics under stress conditions (Zaharieva *et al.*, 2001; Pradhan *et al*., 2012b; Awlachew *et al.*, 2016). Additional tolerance sources have been identified in species such as *Triticum monococcum*, *Aegilops longissima*, and *Aegilops searsii* (Pradhan *et al*., 2012b). Overall, global wheat genetic resources represent a vast but still underutilized reservoir of alleles for heat tolerance, and integrating genomic tools, high-throughput phenotyping, and targeted pre-breeding will be essential to accelerate their deployment in climate-resilient wheat breeding.

## Genomic dissection of heat tolerance

### Genome mapping

Given the limited progress from agronomic management and conventional breeding for thermotolerance, identifying heat resistance genes or QTLs and deploying them in breeding programmes is a priority. In recent years, QTL mapping has been widely used to dissect the genetic basis, environmental interactions, and molecular mechanisms underlying heat stress regulation in wheat (K. Singh *et al*., 2022; http://www.wheatqtldb.net/). A refined list of QTLs with PVE (phenotypic variance explained) >15% from studies over the past decade is provided in Table 1. These studies commonly used recombinant inbred line (RIL) and double haploid (DH) populations and wild relative introgressions for QTL mapping, with some also employing multi-environment trials to validate QTL stability. Several QTLs have already been converted into breeder-friendly KASP (kompetitive allele-specific PCR) markers, enabling marker-assisted recurrent selection and accelerating thermotolerance breeding (Kumar *et al.*, 2024). Such validated loci hold strong promise for improving heat resilience through molecular strategies.

**Table 1.** Details of major QTLs associated with heat stress tolerance in wheat

Traits	Parents	Population number and type	Genotyping platform/marker system	Name of QTLs	Chromosome	Nearest marker and flanking marker	QTL position (cM)	LOD score	PVE (%)	References
Grain weight per spike	HD2808×HUW510	251 RILs	SSR markers	Qgws.iiwbr–2A	2A	Gwm448	171.41	4.45	7.20–20.04	Bhusal *et al.* (2017)
1000-grain weight				Qtgw.iiwbr-2A	2A	Gwm122	168.01	5.65	12.15–30.63	
Grain filling duration				Qhtsigfd.iiwbr–2B	2B	Gwm257	28.01	3.74	15.23	
Grain weight per spike				Qtgws.iiwbr-2A	2A	Gwm497.1	41.61	4.73	28.85	
TS CHCDAA (chlorophyll content at anthesis	HD2808×HUW510	251 RILs	SSR markers	Qtchca.iiwbr-2A	2A	wmc728	123.50	7.10	17.96–28.56	Bhusal *et al.* (2018)
TS CHC15DAA (chlorophyll content 15 d after anthesis)				Qchc.iiwbr-2A	2A	gwm372	149.50	7.07	6.98–15.38	
TS Fm 7 DAA (maximum fluorescence 7 d after anthesis)				Qfm.iiwbr-2D	2D	barc228	104.90	2.46	4.61–16.55	
Grain number per main spike-HS	Hanxuan 10×Lumai 14	150 DH lines	SNP and SSR markers	QGNP-HS1	4D	AX-89703298–AX-89421921	14.80–18.80	13.48	10.03–18.36	Li *et al.* (2019)
Grain yield per plant-HS				QGYP-HS1	1B	AX-111125138–AX-95194017	100.50–110.80	9.41	22.55	
Grain number per main spike-HS-R				QGNP-HS-R1	1A	AX-95652063–AX-95660318	98.3–99.2	20.41	11.13–24.51	
				QGNP-HS-R6	6B	AX-95177681–AX-94427873	87.4–87.5	17.52	4.08–29.32	
1000-kernel weight-HS-R				QTKW-HS-R3	5A	AX-111764369–AX-95659703	62.00–63.40	10.59	11.97–19.51	
Grain yield per plant-HS-R				QGYP-HS-R1	1A	AX-111105973–AX-94402739	81.20–82.50	13.50	20.96	
Plant height	WH1021×WH711	80 RILs	SSR markers	Qph.ccshau-2A	2A	xgwm512–xgwm448	35.30	2.10	17.74	Sangwan *et al.* (2019)
Plant height				Qph.ccshau-4A	4A	xgwm165–xcfd71	25.10	3.20	33.37	
Days to heading				Qdh.ccshau-2A	2A	xgwm512–xgwm448	35.30	2.70	38.11	
Intrinsic water use efficiency				Qiwu.ccshau-2.3A	3A	xgwm512–xgwm448	35.30	10.00	48	
Photosynthetic rate				Qpn.ccshau-2.1D	1D	barc124–xgwm102	27.90	3.60	78.13	
Days to heading	HD2733×WH730	134 BILs	SSR markers	qDH_iari_5A	5A	Xbarc186–Xbarc151	116.0	7.11	23.15	Raveendran *et al.* (2020)
Anthesis date	MACE×GLADIUS, SCOUT×MACE, SCOUT×GLADIUS, RAC1548×GLADIUS, AUS17750×GLADIUS, AUS17840×GLADIUS, HALBERD×KENNEDY	7 DH	SNP markers	QFlt.agt-SG.2B	2B	AXAFFSNP-00483	34.30–38	34.27	25.60	Telfer *et al.* (2021)
Anthesis date				QFlt.agt-MG.2B	2B	AXAFFSNP-07369	34.30–37.70	10.56	28.40	
Anthesis date				QFlt.agt-RG.2B	2B	AXAFFSNP-07428	37.30–42	43.72	83.00	
1000-kernel weight	Vida×MTHW0202	155 RILs	Illumina iSelect 90K SNP array	QTgw.4A	4A	IWB75315	94	10.30	16.30	Cook *et al.* (2021)
Seed diameter				QSd.3A	3A	IWB8648	28	7.00	16.70	
Seed number per head				QSNPH.4A	4A	IWB57145	92.30	11.70	21.40	
Heading date				QHd.4A	4A	IWB59451	90	11.60	15.10	
Heading date				QHd.6B	6B	IWB73728	47	12.90	17.10	
Number of germinated seeds	Zagros × Gonbad, Kuhdasht × Gonbad	120 RILs	SSR markers	qNGS-1A	1A	Xwmc744-1A- cfa2219	102.29	3.42	22.15	Sabouri *et al.* (2022)
Flag leaf length				qFLL-1Da	1D	BARC169- Xwmc147-1D	70.51	3.86	17.62	
Grain length				qGL-4D	4D	BARC48-BARC288	196.25	2.79	27.98	
1000-grain weight				qTGW-1D	1D	Xgpw7258-1A- BARC287	177.28	3.10	22.54	
Number of tillers				qNT-2B	2B	BARC00-gwm429	37.23	3.12	27.64	
Spike length				qSPL-7B	7B	Xwmc335-7B-gwm302	127.75	2.92	26.06	
Grain spike weight				qGWSP-7A	7A	cfa2257- cfa2257	121.47	2.54	21.36	
Leaf senescence	HD3086 X HI1500	166 RILs	35 K SNP	QLSR.iari.4 B	4B	AX94957045–AX95215762	335	5.16	15.33	Taria *et al.* (2023)
Stress tolerance indices-1000-grain weight					3A	AMP0014988–AMP0016989	129	18.63	15.53	
Grain yield	PBW343×KSG1203	177 DH Lines	SNP markers	QGy.ccsu-6B	6B	SNP21108_297—SNP20304_1221	142.90–145.90	5.70	9.20–21.80	Kumar *et al.* (2024)
Number of tillers				QNt.ccsu-1A	1A	SNP897_825 - SNP936_962	487.5–487	5.50	8.20–15.50	

Unlike biparental mapping, GWAS captures broader allelic diversity and offers higher resolution (Saini *et al.*, 2022), making it especially valuable for breeding heat-resilient cultivars. GWAS has been applied extensively to identify loci associated with heat tolerance across wheat developmental stages and trait categories. Trait-focused GWAS on parameters such as CTD, stay-green, and membrane stability not only strengthen genotype–function relationships but also reveal that most marker–trait associations (MTAs) explain only a modest fraction of phenotypic variance (Pradhan *et al.*, 2020; Khan *et al.*, 2024). To ensure robustness, only MTAs from the past decade meeting Bonferroni or false discovery rate (FDR) thresholds are listed in Table 2. Key findings include: (i) pleiotropic loci linking physiological and yield traits; (ii) environmentally stable MTAs with strong breeding relevance; and (iii) the underutilized D genome and synthetic or wild-derived germplasm as reservoirs of novel alleles.

**Table 2.** Details of major marker trait associations (MTAs) associated with heat stress tolerance in wheat

Trait	Population	Genotyping platform	Chromosome	Number of stable MTAs	Reference
Days to heading/days to maturity	188 diverse ICARDA bread wheat	1785 DArT® markers	7A	1	Ogbonnaya *et al.* (2017)
Days to heading			6A,5A,5B,5D	4	
Grain filling duration			1B	1	
Grain weight per spike			1A,4A	2	
Grain yield			1,2B,6B	3	
Kernels per spike			2D,7A	2	
Leaf rolling			2B	2	
Number of spikes in 1 m^2^ (SM^−2^), spikelets per spike			1A,3B,4B,1D,5A,5D,7A,7B,7D	8	
1000-kernel weight			1A,1B,2D,5A,6A	5	
Awn length	108 diverse wheat panel	15 k Illumina SNP	7D	5	Qaseem *et al.* (2018)
Biomass			2A,2B,3B	5	
Days to anthesis			3B,3D,	4	
Days to heading			2A,5A,7D	8	
Grains per spike			2A,5A,7D,	9	
Grain yield			2B,3B,4B,3D	12	
Peduncle extrusion			1B,2B,3B,7D	16	
Plant height			7D	6	
Peduncle length			1B,3B,5A	10	
Spike length			5A	3	
Tillers per plant			2B,3B,3D,4B	12	
Percent reduction			2B,5D,5A	5	
Biomass	295 diverse advanced lines	35 K Axiom® Wheat Breeder's Array	6B	1	Devate *et al.* (2022)
Canopy temperature			7D	1	
Days to heading			1D,2D,2B,3A,5D,6D	18	
Days to maturity			2D,5B,6D	6	
Grain weight per spike			2A,2B,3A,5B,7B	5	
Normalized Difference Vegetation Index at anthesis			2A,2B,3B,2D,1D,1A,5B	7	
Normalized Difference Vegetation Index at 10 d after anthesis			2B,4A,4B	4	
Normalized Difference Vegetation Index at 20 d after anthesis			5A,5B,7A	3	
Plant height			5B,6B	2	
Days to heading	420 Ethiopian durum wheat landraces	Illumina Infinium 25k wheat SNP	1B,2A,5B,6B,7A,7B	6	Mulugeta *et al.* (2023)
Days to maturity			2A,6A,7A	4	
Grain-filling period			3B,6B	2	
Grain yield			1B,5A,7A	4	
Number of effective tillers per plant			1B,7B	2	
Plant height			1B,5A,5B,6B,7B	7	
Spike length			1B,2A,5A	5	
Spikelets per spike			1B,2B,3A,4A,7A	7	
1000-grain weight			1B,3B,5A,6A,7A	7	
Transpiration rate at vegetative stage	158 wheat accessions from Pakistan	SSR markers	1B,3A,3B,4D	4	Khan *et al.* (2024)
Transpiration rate at reproductive stage			1A,1B,3A,6D,7A	5	
Canopy temperature depression at reproductive stage			1A,1B,3A,2B,3B,6A	7	
Photosynthetic rate at vegetative stage			3A,5B	2	
Stay-green			7A	1	
Grain yield			1A,1B,3D,5A,5B	11	
Cell membrane injury at vegetative stage			4B,4D	3	
Photosynthetic rate at reproductive stage			3A,5B	3	
Canopy temperature at 50% anthesis	278 advanced breeding lines	35 K SNP Axiom array	2B,5A,7B	3	Malakondaiah *et al.* (2025)
Canopy temperature at 10 d after anthesis			2D	1	
Canopy temperature at 20 d after anthesis			1D,4A,7A	3	
Normalized Difference Vegetation Index 10 d after anthesis			7D	2	
Normalized Difference Vegetation Index 20 d after anthesis			1A,5D,7D	3	
Soil and plant analysis development 10 d after anthesis			4A	1	
Stress susceptibility index			2B	1	
Magnesium	145 multiple synthetic derivatives	DarT 90 942 SNP markers	1B,3D,4D	3	Emam *et al.* (2025)
Barium			5D	34	
Phosphorus			4D	2	
STI-1000-grain weight			4D,5D	4	

Despite these advances, mapping of heat tolerance traits remain limited. Many QTLs and MTAs have small effects or are environment specific, restricting their utility for marker-assisted selection (MAS). Functional validation is rare, and most studies examine heat stress in isolation rather than the combined stresses typical of production systems. Greater use of meta-QTL/meta-GWAS, haplotype-based mapping, and integration with pangenomic and transcriptomic resources, together with multi-environment trials and broader use of diverse germplasm, particularly from the D genome, will be essential to identify stable loci and accelerate breeding for durable heat resilience.

### Candidate gene mining

Genetic studies have identified numerous genes contributing to wheat heat stress tolerance (Kaur *et al.*, 2019). Many encode regulatory proteins central to heat shock signalling, including heat shock transcription factor A2d (*HsfA2d*), *HSF3*, *HsfA6f*, *HsfC2a*, heat shock protein 16.9 (Hsp16.9), and small heat shock protein 26 (sHsp26) (Comastri *et al.*, 2018; Agarwal and Khurana, 2019; Duan *et al.*, 2019; Liu and He, 2020), while others encode protective proteins with diverse stress-related functions (Agarwal and Khurana, 2018; Li *et al.*, 2018; Tian *et al.*, 2019). Trait-linked genomic analyses have further refined candidates: Malakondaiah *et al.* (2025) identified UDP-glycosyltransferase C4-like and detoxification-related proteins associated with SPAD and canopy temperature, while *in silico* expression profiling by Taria *et al.* (2025) highlighted sugar transporter *MST4*, PPR proteins, trehalose phosphate synthase, and NRT1/PTR FAMILY 8.3-like proteins linked to stem density under heat. Meta-QTL mapping integrated with the wheat reference genome revealed numerous high-confidence candidates, including 102 protein kinases, 55 F-box proteins, 43 cytochrome P450s, 38 leucine-rich repeats, 37 zinc finger proteins, and 27 pentatricopeptide repeat proteins (Kumar *et al.*, 2021).

Pathway-focused and transcriptomic studies have expanded the list. Su *et al.* (2019) identified flavonoid biosynthesis-related protein *(TaFBR1), TaFBR2* and *TaFBR3*, homologous to Arabidopsis *C4H*, *FLS1*, and *TT5* in the flavonoid biosynthesis pathway, as potential regulators of heat tolerance. Kumar *et al.* (2024) reported 209 differentially expressed genes (DEGs) from 28 families, with examples including *ELF3-A1* (earliness), *HSFA1-B1* (thermotolerance), and *VIN2-A1* (grain size) (H. Wang *et al*., 2023). In a QTL-based study, Krishnan *et al.* (2024) shortlisted 21 candidates such as cytochrome P450s, lectin receptor kinases, splicing factors, ABC transporters, alcohol dehydrogenases, F-box proteins, plastid-lipid proteins, and MYB TFs. More recently, Deng *et al.* (2025) characterized *COBL6A2* as a key regulator and promising breeding target.

Heat tolerance in wheat involves interactions among regulatory, structural, and metabolic genes affecting photosynthetic stability, reproductive protection, and yield maintenance. Although multi-omics and QTL-based studies have identified many candidate genes, functional validation remains limited. Their stage-specific roles, particularly during reproduction and grain filling, and integration into regulatory networks are still poorly understood. Future research should focus on field validation, allele mining, and gene editing to translate these candidates into breeding applications.

## Advanced molecular strategies

### Multiomics and the role of transcription factors and co-activators in heat tolerance

High-throughput multiomics technologies have advanced the understanding of heat tolerance by providing system-level insights into molecular responses. In wheat, transcriptomic studies using microarrays and RNA-seq show widespread reprogramming under heat stress, affecting protein folding, hormone signalling, and metabolic regulation (Qin *et al.*, 2008; Zheng *et al.*, 2025). Proteomic analyses complement these findings, highlighting discrepancies between mRNA and protein abundance; for instance, Zhang *et al.* (2017) identified >250 heat-responsive proteins, underscoring extensive post-transcriptional regulation. Metabolomic profiling has revealed shifts in sugars, amino acids, and antioxidants, reflecting adaptive roles in osmotic balance, ROS detoxification, and energy homeostasis (Sehgal *et al.*, 2023). Integrating transcriptomic, proteomic, metabolomic, hormonal, and physiological data helps identify regulatory hierarchies and hub genes. Da Ros *et al.* (2023), for instance, combined multi-stress datasets and identified 152 hub genes, including *WRKY33*, emphasizing the value of systems biology for uncovering regulators and guiding gene-stacking strategies for thermotolerance.

Among the most important regulators revealed are TFs, which orchestrate large gene networks in response to heat (Guo *et al.*, 2016). Recurrently implicated families include HSFs, DREBs, bZIPs, NACs, and MYBs. Class A HSF genes such as *HsfA2* and *HsfA6* activate HSPs, including HSP70s and sHSPs, which refold denatured proteins and prevent aggregation (Xue *et al.*, 2014). Overexpression of *HsfA6f* improves thermotolerance in model systems (Bi *et al.*, 2020). DREB and NAC TF genes such as *NAC2L* regulate heat-responsive genes through hormone crosstalk (Guo *et al.*, 2015). Activation of the IRE1 pathway splices *bZIP60* into its functional form, enhancing the unfolded protein response (Geng *et al.*, 2018). Several heat-responsive MYB genes have also been identified, offering potential for MAS (Magar *et al.*, 2025).

TF activity is often modulated by co-activators, non-DNA-binding proteins that bridge TFs and the transcriptional machinery. Only class A HSFs possess the C-terminal AHA (aromatic, hydrophobic, acidic) activation domain, enabling co-activator function, while classes B and C generally lack this motif and act as repressors (Meng *et al.*, 2022). Functional studies highlight this distinction: *HsfA6f* activates HSPs, GAAPs, and Rubisco activase (RCA) (Xue *et al.*, 2015), while *HsfA2-13* has been reported to acts as a co-activator, though validation is needed (Meng *et al.*, 2022). Post-translational modifications also influence coactivator activity; H. Wang *et al*. (2023) showed that SUMOylation enhances *HsfA1* activity but declines under prolonged stress, down-regulating the response. *HsfA1* also interacts with histone acetyltransferase *HAG1* in a temperature-sensitive manner, functioning as an ON/OFF transcriptional switch. Alternative splicing further diversifies co-activator function: HSFA6e isoforms differ in activity, with the tryptophan-rich AHA domain in HSFA6e-III enabling stronger activation of *HSP70* genes (Wen *et al.*, 2023).

Beyond HSFs, other co-activators are important. *MBF1c* overexpression in rice improved heat tolerance by up-regulating HSPs and trehalose phosphate synthase genes (Qin *et al.*, 2015). In wheat, *MBF1c* is heat inducible, localizes to stress granules, and activates stress-responsive genes (Tian *et al.*, 2022). It bridges TFs such as GCN4, FTZ-F1, and ATF1 with the TATA-box binding protein, integrating developmental and stress programmes. Wheat DREB genes, including *DREB2B* and *DREB6A*, are also up-regulated under heat and may activate HsfA3-like pathways (Qin *et al.*, 2008). MBF1 family co-activators additionally respond to ethylene signalling during heat stress, suggesting a coordinated TF–co-activator module that enhances thermotolerance.

Collectively, multiomics studies show that wheat heat tolerance is governed by TF–co-activator networks regulating protective proteins, ROS detoxification, osmolyte accumulation, and hormonal signalling. Although regulators such as *HsfA6f*, *NAC2L*, and *MBF1c* show functional relevance, many remain poorly characterized under field-relevant heat stress. Future work should prioritize functional validation, regulatory network mapping, and allele mining to enable deployment of key regulators for breeding heat-resilient wheat cultivars.

### Epigenetic mechanisms and stress memory in heat tolerance

**Fig. 3.**
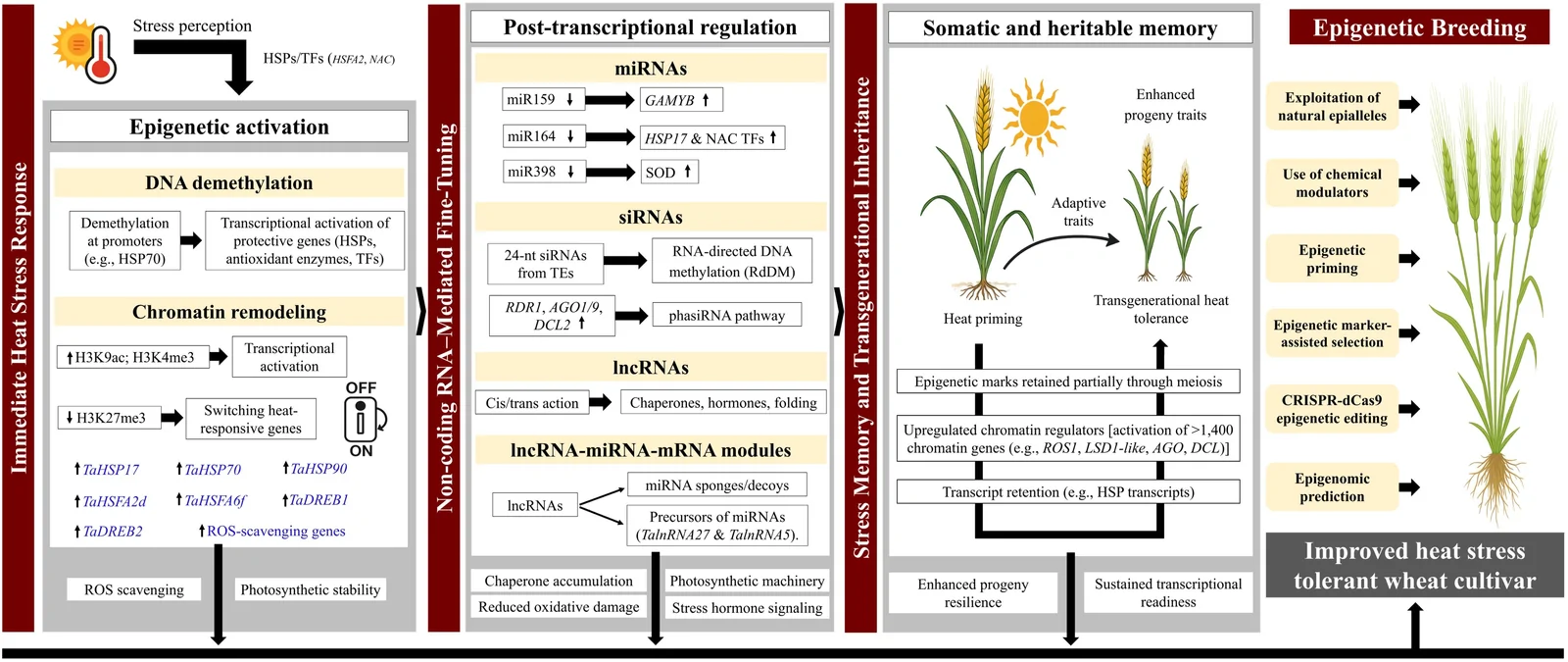
Conceptual framework linking immediate heat stress responses, epigenetic regulation, transgenerational memory, and breeding strategies for developing heat stress-tolerant wheat. Upon heat perception, transcription factors (e.g. HSFs and NACs) initiate epigenetic activation through DNA demethylation and chromatin remodelling (increased H3K9ac, H3K4me3; reduced H3K27me3), leading to rapid transcription of heat-protective genes such as HSPs, DREBs, and ROS scavengers. These processes are further fine-tuned post-transcriptionally by small RNAs (miRNAs and siRNAs) and lncRNAs that act via transcript cleavage, sponging, folding control, or RdDM. Heat priming induces somatic and heritable epigenetic memory, characterized by transcript retention (e.g. HSPs) and persistent activation of chromatin regulators (e.g. ROS1, DCLs, and LSD1), which are partially retained through meiosis. These mechanisms inform epigenetic breeding strategies, including the exploitation of natural epialleles, chemical modulators, epigenetic priming, marker-assisted selection, CRISPR/dCas9 epigenome editing, and predictive modelling, facilitating the development of heat-resistant wheat cultivars. Created in BioRender. Chitikineni, A. (2026) https://BioRender.com/1kwndjj.

Epigenetic modifications complement genetic factors by dynamically regulating gene expression under heat stress through DNA methylation, histone modifications, chromatin remodelling, and non-coding RNAs. Some marks persist across cell divisions or are inherited, offering potential for heritable stress resilience (Fig. 3).

#### DNA methylation dynamics and histone modifications under heat stress

DNA methylation and histone modifications are the most studied epigenetic marks in wheat, shaping gene expression and genome stability during stress. The polyploid genome exhibits high baseline methylation (>40–50%) across CG, CHG, and CHH contexts (Gardiner *et al.*, 2015). Heat stress induces modest but targeted changes, affecting ∼0.1% of loci under mild stress (27 °C), with differentially methylated regions often near heat-responsive genes (Gardiner *et al.*, 2015). Hypomethylation in gene-rich regions can derepress stress-inducible genes or activate transposable elements, though large-scale mobilization has not been reported in wheat (Liu and He, 2020; Sun *et al.*, 2022). Active demethylation appears adaptive, supported by up-regulation of ROS1-like and LSD1 (lysine-specific histone demethylase 1A)-like demethylases (X. Wang *et al.*, 2016; Gahlaut *et al.*, 2022) and down-regulation of maintenance methyltransferases in other species. Such adjustments may facilitate heat-induced gene activation and contribute to stress memory (Fig. 3).

In addition to DNA methylation, histone modifications regulate chromatin accessibility and transcriptional responses to heat stress. Wheat encodes >245 histone-modifying enzymes (Zheng *et al.*, 2021), many of which show heat-responsive expression patterns. Activating histone marks such as H3K9 acetylation accumulate at promoters of heat-shock genes, including HSP70 in *A. thaliana* (Nishio *et al.*, 2024), while persistent H3K4me3 may function as a chromatin ‘bookmark’, enabling rapid transcriptional reactivation during recurrent heat stress (Kappel *et al.*, 2023). Conversely, repressive marks such as H3K27me3 are often reduced at heat-inducible loci, thereby facilitating transcriptional activation. Evidence for the functional importance of histone modifications in wheat is illustrated by *HAG1*, a wheat homologue of the Arabidopsis histone acetyltransferase gene *GCN5*. HAG1 interacts with the TF NAC1 to activate downstream genes such as *G1* and *PSBR1*, which stabilize PSII and enhance thermotolerance. Overexpression of *HAG1* improves heat resilience, whereas knockout mutants exhibit increased heat sensitivity, highlighting its potential as a target for breeding heat-tolerant wheat cultivars (Lin *et al.*, 2022).

#### Non-coding RNAs in heat stress regulation

Non-coding RNAs are pivotal regulators of gene expression and epigenetic control. In wheat, miRNAs, siRNAs, and lncRNAs coordinate transcriptional reprogramming and post-transcriptional regulation during heat stress (Fig. 3).

miRNAs, ∼21 nt in length, silence genes through mRNA cleavage or translational repression. High-throughput sequencing identified >202 miRNAs under heat stress, with 36 differentially expressed, targeting 589 transcripts linked to stress signalling, antioxidant defence, and protein modification (Ravichandran *et al.*, 2019). Conserved miRNAs such as miR156, miR166, and miR393 regulate TFs including SPLs, HD-ZIPs, and TIRs, while tae-miR9775 and tae-miR1120a are up-regulated in tolerant genotypes (Saroha *et al.*, 2024). Functional studies show that miR159 down-regulation permits GAMYB TF accumulation and tolerance, whereas its overexpression suppresses GAMYB and reduces resilience (Wang *et al.*, 2012). Similarly, repression of miR164 enables *HSP17* accumulation and NAC-mediated defence (Kumar *et al.*, 2015). miR398, targeting Cu/Zn-superoxide dismutase (SOD), is transiently suppressed in Arabidopsis to enhance ROS scavenging (Li *et al.*, 2022) and probably functions similarly in wheat. Other heat-responsive miRNAs such as miR156 and miR171 integrate developmental and stress pathways (Zhao *et al.*, 2016).

siRNAs, often derived from transposons, act in post-transcriptional silencing and RNA-directed DNA methylation (RdDM). In Arabidopsis, RdDM components Pol IV and Pol V are essential for transposon silencing and thermotolerance, with mutants showing impaired stress memory (Talarico *et al.*, 2024). In the transposon-rich genome of wheat (>80% repetitive), 24 nt siRNAs predominantly target transposons (Y. Wang *et al.*, 2016). Heat-induced transposon activation may trigger siRNA production, reinforcing silencing via feedback loops, while secondary siRNAs can modulate stress-responsive genes. Transcriptomic evidence shows that AGO1, AGO9, and DCL2 are constitutively expressed under control and stress, while RDR1 is heat induced in leaves, suggesting roles for phased siRNAs and RDR6-dependent species (R.K. Singh *et al*., 2022).

lncRNAs, having transcripts >200 nt, regulate gene expression at transcriptional and post-transcriptional levels. In developing pollen, Babaei *et al.* (2024) identified 11 054 lncRNAs, of which 5482 were differentially expressed under heat, implicating them in pollen thermotolerance. Many act *in cis* or *in trans*, targeting hormone signalling, protein folding, and stress responses, and show associations with HSP genes. Some function as miRNA precursors (e.g. *lnRNA27* and *lnRNA5*) or as sponges sequestering miRNAs to prevent repression (Xin *et al.*, 2010). Although many require validation, their dynamic, stress-specific expression highlights their role in fine-tuning the heat response in wheat.

#### Epigenetic stress memory and transgenerational inheritance

Stress-induced epigenetic changes may contribute to molecular memory that influences responses to recurring stress. This memory may be somatic, persisting within an individual, or transgenerational, transmitted to offspring. Somatic memory has been associated with sustained activation of TFs such as HSFA2, persistence of histone marks such as H3K4me3, and retention of stress-responsive transcripts (Xue *et al.*, 2014; Friedrich *et al.*, 2021). In wheat, mild heat priming during grain filling has been reported to enhance progeny thermotolerance, improving yield, photosynthesis, and antioxidant capacity even without direct stress exposure (X. Wang *et al.*, 2016). Multi-omics profiling of primed plants revealed >1400 DEGs, including chromatin regulators, suggesting possible involvement of epigenetic regulatory pathways. Evidence for transgenerational inheritance is also emerging: Liu *et al.* (2021) reported that parental heat stress improved offspring traits, with 1771 miRNAs and >66 000 DEGs enriched in hormone signalling and stress-related pathways. Although most epigenetic marks are reset during reproduction, stress events near gametogenesis or seed development may escape complete resetting, potentially enabling partial inheritance of stress-associated states.

Epigenetic mechanisms in wheat, including DNA methylation, histone modifications, and non-coding RNA networks, are associated with heat-stress responses and may influence transcriptional regulation and stress memory. However, their direct role in stable thermotolerance remains unclear. Key challenges include field validation, resolving homeologue-specific variation, integrating multi-omics datasets, and determining whether primed states consistently improve yield under recurrent heat stress.

## Genomic technologies for breeding applications

### Genome sequencing and pangenomics

Advances in next-generation sequencing (NGS) have transformed wheat genomics, enabling high-quality reference genomes and large-scale exploration of diversity (Della Coletta *et al.*, 2021; Y. Wang *et al*., 2023). Bread wheat, an allohexaploid with a complex and expansive genome (∼17 Gb), poses major assembly challenges. A milestone was the release of the Chinese Spring reference genome by the International Wheat Genome Sequencing Consortium (IWGSC) in 2018, generated with chromosome-arm-specific shotgun sequencing (Tiwari *et al.*, 2024). This reference underpins genome-assisted breeding, trait dissection, and gene discovery, including genes for heat tolerance. Whole-genome sequencing (WGS) has accelerated the discovery of SNPs, InDels, and structural variants (SVs) in heat-responsive genes and regulatory elements (Rehman *et al.*, 2022). For instance, re-sequencing of *T. sphaerococcum* identified *SG-D1*, a kinase gene enhancing thermotolerance by stabilizing PIF4, which activates HSPs and ROS-scavenging pathways. In contrast, deleterious InDels in the *PIF4* promoter, common in modern Chinese cultivars due to European introgressions, reduce heat tolerance (Cao *et al.*, 2024). Such findings show how sequencing reveals both beneficial alleles and genetic bottlenecks. High-quality assemblies also improve the resolution of QTL mapping and GWAS, refining the identification of loci for thermotolerance (Lafarge *et al.*, 2017).

A single reference genome, however, fails to capture full intraspecific variation. Pan-genomics addresses this by cataloguing both core (shared) and dispensable (variable) genes across diverse genotypes (Sarawad *et al.*, 2025). Pangenomes built from modern cultivars, landraces, and wild relatives uncover novel genes and alleles absent in the reference but associated with crucial processes, including protein homeostasis, signal transduction, membrane integrity, and antioxidant defence (Raza *et al.*, 2026). Whole-genome-assembly-based pangenomes now exist for rice (Zhou *et al.*, 2020), barley (Jayakodi *et al.*, 2020), wheat (Walkowiak *et al.*, 2020), maize (Hirsch *et al.*, 2014), soybean (Li *et al.*, 2014), and brassica (Song *et al.*, 2020).

The wheat pangenome has revealed extensive SVs, presence–absence variants (PAVs), and novel genes linked to agronomic and stress-adaptive traits (Arif *et al.*, 2025). Walkowiak *et al.* (2020) characterized SVs and non-reference genes across multiple cultivars, while Montenegro *et al.* (2017) identified agronomically important loci via pangenomic comparisons. In pearl millet, Yan *et al.* (2023) found SVs near endoplasmic reticulum (ER)-stress genes and RWP-RK TFs associated with heat tolerance; similar variants in wheat may affect photosynthesis and membrane stability. In rice, a pangenome of 60 heat-tolerant varieties revealed 1141 non-reference genes, of which were 24 heat inducible (Woldegiorgis *et al.*, 2022). Likewise, wheat pangenomes uncovered missing genes in modern cultivars that persist in wild emmer and *Aegilops* species, providing valuable resources.

Integrating pangenomic data with transcriptomic, epigenomic, and proteomic analyses offers deeper mechanistic insights. Pan-transcriptomics identifies DEGs in dispensable regions, revealing genotype-specific stress responses (Bhatti *et al.*, 2020). In wheat, SVs near *HSP17.1* correlate with higher expression under heat, while PAVs in *HSFA6f* promoters alter TF-binding sites (Xue *et al.*, 2015). Pangenomic studies have also uncovered chromatin remodellers and non-coding RNAs associated with SVs (Raza *et al.*, 2023). A notable example is a 12 kb insertion at the *HST1* locus in Pakistani landraces, coinciding with DNA methylation changes that enhance transcription under heat stress (Khan *et al.*, 2022). Beyond discovery, pangenomic resources support high-resolution marker development, genomic prediction, and identification of rare haplotypes for MAS and genome editing. Including diverse germplasm, especially *T. dicoccoides* and *T. urartu*, further expands the genetic base for heat resilience (Yao *et al.*, 2025).

Despite these advances, major challenges remain. Many dispensable and non-reference genes associated with heat tolerance are still uncharacterized, and SVs and PAVs often lack functional validation. Current wheat pangenomes also under-represent diversity from key heat-adapted regions such as South Asia, Africa, and the Middle East. Expanding pangenomic datasets, validating candidate genes under field conditions, integrating multi-omics layers, and incorporating these resources into predictive breeding and genome editing pipelines will be essential to accelerate the development of heat-resilient wheat cultivars.

### CRISPR/Cas-based targeted gene editing

The CRISPR/Cas system has revolutionized plant genetics by enabling precise, efficient editing even in complex polyploids such as wheat. Since its first application in 2013 (Shan *et al.*, 2013), >80 genes have been edited for diverse traits (Zhou *et al.*, 2023). For heat tolerance, CRISPR is used both to validate candidate genes and to create stress-resilient alleles. A clear example of functional genomics is the knockout of *DMC1*, essential for meiotic chromosome pairing. Wheat meiosis is highly temperature sensitive, and CRISPR mutants lacking *DMC1* showed severe crossover loss and sterility at both low (13 °C) and high (30 °C) temperatures, confirming its role in reproductive resilience (Draeger *et al.*, 2023). Beyond validation, CRISPR enables engineering of heat-tolerant alleles: Li’s group at SDSU is editing RCA to enhance thermostability and sustain photosynthesis (Nagarajan *et al.*, 2025). Likewise, the beneficial *E286K* allele of *SG-D1*, which stabilizes PIF4 to activate HSP and ROS pathways, can be introduced into elite cultivars without linkage drag (Cao *et al.*, 2024).

CRISPR can also target negative regulators; for instance, knocking out wheat homologues of Arabidopsis *BZR1* could enhance heat tolerance, though yield penalties must be avoided. Emerging base and prime editors enable subtle promoter or coding modifications that fine-tune gene expression, particularly valuable for SNP-driven or weak alleles. Editing in wheat remains challenging since all three homeologues often require modification, but multiplexed guide RNAs and improved transformation systems, including callus-based protocols and viral guide delivery, are addressing this. Notably, applying a heat pulse during tissue culture increased editing efficiency (Kishi-Kaboshi *et al.*, 2023), suggesting that stress itself may enhance resilience engineering. Despite these advances, CRISPR applications for heat tolerance remain limited. Most studies target single genes, whereas thermotolerance is polygenic and requires network-level editing. Few edited lines have been tested under field conditions, and stability across environments remains uncertain. Future work should focus on multiplex editing of thermotolerance genes, validation of pangenome- and GWAS-derived candidates, testing under diverse heat regimes, and integration with genomic selection to accelerate breeding.

### Genomic prediction and haplotype-based breeding

Breeding for heat tolerance in wheat is difficult due to quantitative genetic control, strong environmental interactions, and yield trade-offs. Conventional phenotypic selection requires costly, multiyear trials that often fail to capture rare heat events. Genomic prediction (GP) offers a faster, cost-effective alternative by estimating breeding values from genome-wide SNPs, capturing small-effect QTLs often missed by MAS. Prediction accuracy depends on training size, marker density, trait architecture, and modelling of G×E interactions, and improves with covariates such as heat degree days and multi-environment models. McBreen *et al.* (2025) showed that integrating SNPs with unmanned aerial vehicle (UAV)-based hyperspectral imaging (HSI) and environmental data improved yield prediction under heat stress (*r* ∼0.53) relative to SNP-only models, as HSI captures physiological traits such as chlorophyll and canopy water content. Similarly, Shahi *et al.* (2024) reported GP accuracies up to 0.60 under post-anthesis heat, with within-environment models performing best.

At CIMMYT, long-term datasets have improved reliability of GP for heat tolerance in South Asia: yield prediction under late heat rose from 0.11 with 1 year of data to 0.26 with 5 years (Vitale *et al.*, 2025). Artificial intelligence-based models such as WheatGP (Wang *et al.*, 2025), which integrates convolutional neural network (CNN) with long short-term memory (LSTM) architectures, achieved accuracies up to 0.73 by capturing both additive and epistatic effects. Other machine learning approaches, including gradient boosting and deep neural networks, outperform traditional models in modelling non-linear interactions (Gao *et al.*, 2025). Reaction-norm models incorporating environmental indices further enhance cross-environment predictions (Montesinos-López *et al.*, 2023, 2024). Despite progress, GP for heat tolerance remains underused. Its integration with speed breeding could accelerate allele stacking, while targeted field screening (e.g. late planting to impose heat stress) and CRISPR-based editing could link predictions with functional validation.

While GP predicts breeding values, haplotype-based breeding tracks favourable allele combinations across multiple loci. Owing to large haplotype blocks of wheat, haplotype-based GWAS often captures causal variation more effectively than SNP analyses. Brinton *et al.* (2020) found that modern cultivars share ∼59% of their genome in identical-by-state haplotypes, yet many beneficial haplotypes remain in landraces. For heat tolerance, Zhai *et al.* (2021) identified 15 haplotypes at the *HST1* locus on 4AL: the tolerant Hap1 retained an intact genomic region, while the globally common Hap2 carried deletions linked to sensitivity. Diagnostic PCR markers now enable Hap1 reintroduction via haplotype-assisted breeding. Sehgal *et al.* (2020) used haplotype-based GWAS to detect heat-responsive haplotypes for grain yield missed by SNP-based analyses, while the functional haplotype-GWAS (FH-GWAS) model (Bhat *et al.*, 2021) further improves discovery of functional haplotypes.

Several promising heat-responsive haplotypes have recently been reported. Palermo *et al.* (2024) identified 17 novel haplotypes of *TdHsp26* in durum wheat, with SSD69 showing a distinct oxidative stress response. Cao *et al.* (2024) discovered a favourable *SG-D1* E286K haplotype that stabilizes PIF4 in Indian dwarf wheat, while Deng *et al.* (2025) identified *COBL6A2* Hap1, enhancing antioxidant activity and seedling vigour. Expanding pangenome resources, such as the 1000 wheat exomes (Vasudevan and Cloutier, 2023), supports targeted donor selection. Landraces such as Aragon 03, valued for abiotic stress tolerance (Zheng *et al.*, 2025; Ullah *et al.*, 2022), may harbour resilient haplotypes for introgression. Computational tools can now simulate crosses and predict progeny haplotype assemblies, streamlining genome design.

Haplotype-based breeding complements GP by precisely tracking multi-gene regions for heat tolerance. Combined with genome editing, these approaches merge predictive accuracy with genetic precision to accelerate climate-resilient cultivar development. Both approaches require refinement: GP needs larger and more diverse training sets under field-relevant heat, integration of physiological traits, and standardized environmental descriptors; haplotype-based breeding requires functional validation, fine-mapping of causal variants, and strategies to minimize linkage drag. Integrating GP, haplotype-assisted selection, speed breeding, and genome editing provides a coherent framework for developing wheat cultivars that sustain yield under rising global temperatures.

## Challenges and future directions

Despite major advances in genomics, phenotyping, and gene discovery, translating wheat heat tolerance research into field-ready cultivars remains slow. Heat tolerance is polygenic, stage-specific, and strongly shaped by G x E interactions, requiring coordinated solutions to overcome current bottlenecks.

- A key limitation is the lack of standardized protocols to distinguish terminal from episodic stress and harmonize indices such as CTD, stay-green, or membrane stability. Reproductive stages remain under-represented, and combined stresses (heat × drought, heat × vapour pressure deficit) are rarely tested. A Heat Phenotyping Standard implemented through multi-environment trials using late sowing, heat tents, or climate-analogue sites, coupled with UAV-based thermal, hyperspectral, and fluorescence sensing, would improve reproducibility and predictive power.
- Many GWAS/QTL signals are population or environment specific, while SVs and PAVs from pangenomes remain underused. Haplotype-based discoveries in diverse germplasm are seldom validated in elite lines. Anchoring association studies to the wheat pangenome, emphasizing cross-population replication, and linking loci to standardized phenotypes with diagnostic markers and isogenic edits will strengthen deployment.
- Multi-omics datasets remain siloed, limiting use in breeding. Critical reproductive-stage networks, including meiosis stability, pollen viability, and early grain filling, are incompletely resolved. Stage-specific regulatory maps integrating tissue-targeted omics, phosphoproteomics, spatial transcriptomics, and dynamic network modelling could identify master regulators for multiplex editing.
- Functional validation lags behind discovery. Editing in hexaploid wheat requires multi-homeologue targeting and is often genotype dependent. A streamlined design–edit–test pipeline, applying base or prime editing and validating under speed breeding followed by multi-environment trials, could accelerate translation.
- GP shows promise but often loses accuracy across environments due to coarse descriptors. Models rarely integrate UAV-derived traits or haplotype/SV features. Reaction-norm GP using fine-scale weather data, UAV phenomics, and genomic priors, linked with speed breeding, could enable faster allele stacking.
- The persistence of heat-induced epigenetic marks in field conditions remains unclear. Standardized priming protocols are lacking, and longitudinal studies are needed to track molecular marks from expression to phenotype across generations. Identifying epiregulatory nodes such as histone acetyltransferases and RdDM components may enable targeted manipulation without yield penalties.
- Progress is slowed by linkage drag, limited seed systems, and fragmented data sharing. Trade-offs such as earliness versus yield are seldom quantified. Haplotype-guided backcrossing, genomic mating, and ideotype-driven breeding that combine canopy cooling, delayed senescence, and reproductive stability can accelerate cultivar development. Coordinated benchmark panels of diverse, elite, and edited lines evaluated in global heat-stress networks with open data sharing will be essential.

In summary, progress will depend on harmonized phenotyping, robust validation of causal variants in elite germplasm, integration of multi-omics into breeding pipelines, and tighter links between discovery and deployment to deliver climate-ready wheat cultivars capable of sustaining yield under intensifying heat stress.

## Conclusion

Heat stress tolerance in wheat results from the coordinated action of physiological, biochemical, and regulatory networks across developmental stages. Advances in trait mapping, transcriptomics, epigenomics, pangenomics, and functional genomics have significantly improved our understanding of thermotolerance in this polyploid crop. Key insights include the importance of reproductive-stage resilience, pleiotropic loci linking yield and physiology, and the growing role of predictive breeding based on genomic and phenotypic data. Integrating multi-omics with high-throughput phenotyping and genome editing has strengthened phenotype-to-gene-to-function links, enabling more targeted crop improvement. Pangenomics continues to reveal novel alleles, while haplotype-based breeding and CRISPR/Cas editing provide powerful tools to accelerate genetic gain. The next challenge is translating these advances into operational breeding pipelines. As climate change intensifies, developing durable heat-resilient wheat cultivars remains both a scientific priority and a societal necessity, requiring close integration of molecular biology, genomics, breeding, and agronomy supported by open data and shared standards.

## Data Availability

This review contains no new experimental data.
